# High-Dimensional Immune Profiling by Mass Cytometry Revealed the Circulating Immune Cell Landscape in Patients With Intracranial Aneurysm

**DOI:** 10.3389/fimmu.2022.922000

**Published:** 2022-06-27

**Authors:** Peicong Ge, Chenglong Liu, Liujia Chan, Yuheng Pang, Hao Li, Qian Zhang, Xun Ye, Jia Wang, Rong Wang, Yan Zhang, Wenjing Wang, Dong Zhang, Jizong Zhao

**Affiliations:** ^1^ Department of Neurosurgery, Beijing Tiantan Hospital, Capital Medical University, Beijing, China; ^2^ China National Clinical Research Center for Neurological Diseases, Beijing, China; ^3^ Center of Stroke, Beijing Institute for Brain Disorders, Beijing, China; ^4^ Beijing Key Laboratory of Translational Medicine for Cerebrovascular Disease, Beijing, China; ^5^ Beijing Translational Engineering Center for 3D Printer in Clinical Neuroscience, Beijing, China; ^6^ Beijing Institute of Hepatology, Beijing YouAn Hospital, Capital Medical University, Beijing, China; ^7^ Department of Neurosurgery, Beijing Hospital, Beijing, China; ^8^ Savaid Medical School, University of Chinese Academy of Sciences, Beijing, China

**Keywords:** intracranial aneurysm, mass cytometry, circulating immune cell, landscape, inflammation

## Abstract

**Background:**

Increasing evidence supports a critical role of chronic inflammation in intracranial aneurysm (IA). Understanding how the immunological alterations in IA provides opportunities for targeted treatment. However, there is a lack of comprehensive and detailed characterization of the changes in circulating immune cells in IA.

**Objective:**

To perform a comprehensive and detailed characterization of the changes in circulating immune cells in patients with IA.

**Methods:**

Peripheral blood mononuclear cell samples from IA patients (n = 26) and age-and sex-matched healthy controls (HCs, n = 20) were analyzed using high dimensional mass cytometry, and the frequency and phenotype of immune cell subtypes were assessed.

**Results:**

We identified 28 cell clusters and found that the immune signature of IA consists of cluster changes. IA patients exhibited dysfunction of immunity, with dysregulation of CD4^+^ T-cell clusters, increased B cells and monocytes, and decreased CD8^+^ T cells, DNT cells, and DPT cells. Moreover, compared with findings in HC, IA was associated with enhanced lymphocyte and monocyte immune activation, with a higher expression of HLA-DR, CXCR3, and CX3CR1. In addition, the expression of TLR4, p-STAT3, and the exhaustion marker PD1 was increased in T cells, B cells, and NK cells in IA patients.

**Conclusions:**

Our data provide an overview of the circulating immune cell landscape of IA patients, and reveal that the dysfunction of circulating immunity may play a potential role in the development of IA.

## Introduction

Intracranial aneurysms (IAs) are an abnormality in the cerebral vasculature with a prevalence of 3.2% among the general population (1). Rupture of an IA can cause subarachnoid hemorrhage, which is a life-threatening acute cerebrovascular event (2, 3). Nearly, one-third of patients die, and another one-third rely on others for performance of daily life activities (4).

To date, although the ultimate causes of IA remain unclear (5), a series of experimental reports and animal studies have highlighted that alterations in the immune system and inflammation may play a crucial role in IA formation (6). Moreover, gene expression analysis of human IA also revealed that multiple proinflammatory genes are upregulated (7). In addition, experimental therapies with different inflammatory molecules in animal models have proven efficacious and promising. IA is increasingly recognized as a disease driven by chronic inflammation (8).

Previous studies have demonstrated that circulating immune cells and molecules contribute to IA formation and rupture (9–11). Whereas, studies regarding specific immune cell classification in peripheral blood mononuclear cells (PBMCs) in IA remain lacking. However, the phenotypic diversity of PBMCs is large, and it is difficult to characterize the changes in all immune cell subsets. Nevertheless, high dimensional mass cytometry, also known as CyTOF, could overcome these deficiencies and has been used to characterize immune populations in the field of vascular diseases (12). Here, we profiled immune cellular components using CyTOF to analyze PBMCs from IA patients by comparing them with those from healthy controls (HCs).

## Materials and Methods

### Subjects

The study was registered and approved by the Ethics Committee of Beijing Tiantan Hospital, Capital Medical University. All the subjects were recruited from the Department of Neurosurgery, Beijing Tiantan Hospital. Patients who met the following criteria were enrolled in this study: 1) patients with at least one IA detected by cerebral angiogram; and 2) patients who had recently experienced trauma or undergone surgery, or had autoimmune diseases, or tumors were excluded. All the patients were newly diagnosed and did not receive any treatment by the time of blood withdrawal. A total of twenty-six patients were enrolled. Twenty age- and sex-matched healthy volunteers with no IA confirmed by magnetic resonance angiography were included in the HC group. All patients and HCs signed written informed consent forms before blood withdrawal.

### Mass Cytometry

Peripheral venous blood samples were extracted into EDTA tubes in the fasting state and processed within 24 hours. PBMCs were isolated by Ficoll-Paque density gradient centrifugation and placed into a −80° C freezer until further analysis. The monoclonal antibodies used in this study are described in detail in **Supplementary Table 1**. According to the manufacturer’s instructions, PBMCs were stained with lanthanide metal-tagged surface antibodies, Briefly, PBMCs were washed with PBS and stained with cisplatin to assess viability. Next, PBMCs were stained with the surface markers. After surface marker staining, the cells were washed. Then, PBMCs were stained with intracellular markers. After intracellular staining, the cells were washed twice before staining in DNA intercalator solution. A Helios mass cytometer (Fluidigm, USA) was used for data acquisition.

### Data Analysis

After preprocessing, the data of single, live cells from each sample were manually gated and exported from Cytobank. The data were then arcsine normalized, for unbiased determination of clusters. Then, PhenoGraph was utilized to analyze the high dimensional data, using the R-package “cytofkit (v1.10.0)” with default parameters (13). The dimensionality reduction algorithm, t-stochastic neighbor embedding (t-SNE) was used to visualize the high-dimensional data in two dimensions and to show the changes in frequency and heterogeneity of cluster and marker expression between two groups. The following parameters were used: perplexity = 45; iterations = 2500; and theta = 0.5. Clusters showing similar phenotypic characteristics were manually merged. Marker expression levels and cell counts in each cluster were exported and statistical analysis was carried out in the R environment. R-package “pheatmap (v1.0.12)” and “ggplot2 (v3.3.5)” were used to plot data. Wilcoxon rank-sum test was used to compare the marker expression levels and cell counts between groups. Two-sided P values of <0.05 were considered statistically significant.

## Results

### Characterization of Circulating Immune Populations

**Figure 1:**
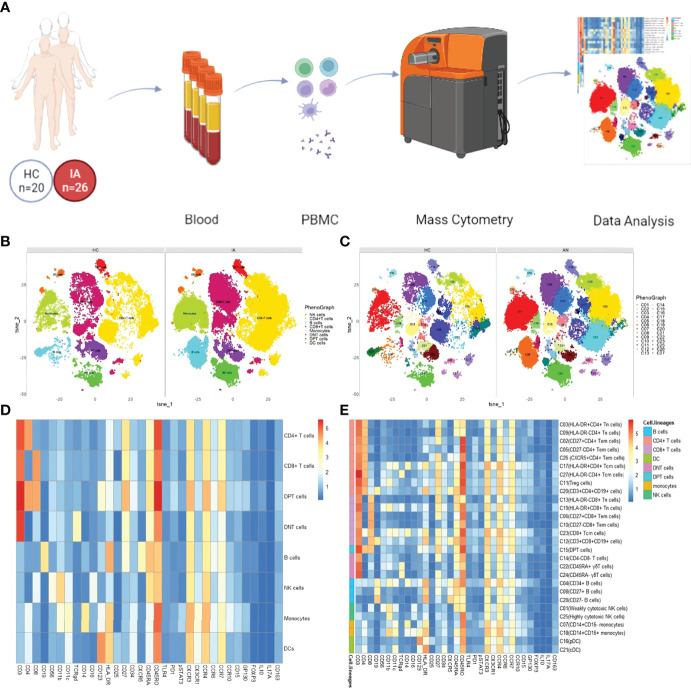
Overview of the imaging mass cytometry study of IA. **(A)** Flowchart overview of PBMC collection. Twenty-six PBMC samples from IA patients were included in this study, as well as 20 control samples. PBMCs were stained with one antibody panels and acquired on the CyTOF instrument. **(B)** t-SNE visualization of CD45+ cells from PBMCs. PhenoGraph clusters analysis of manual gated eight-cell lineages, including CD4^+^ T cells, CD8^+^ T cells, CD4^+^CD8^+^ double-positive T cells (DPT), CD4^-^CD8^-^ double-negative T cells (DNT), B cells, NK cells, dendritic cells (DCs), and monocytes. **(C)** Representative t-SNE plot of 28 cell clusters from HC and IA patients:CD4^+^ T cells, nine clusters (C02, C03, C05, C09, C11, C17, C20, C26, C27); CD8+T cells, six clusters (C06, C10, C12, C13, C19, C23); DPT, one cluster (C15); DNT, three clusters (C14, C22, C24); B cells, three clusters (C04, C08, C28); NK cells, two clusters (C01,C25); monocytes, two clusters (C07, C18); DC, two clusters (C16, C21) **(D)** Heatmap showing median marker expression across the 8 cell lineages **(E)** Heatmap of the expression of each protein in 28 cell clusters.

**Figure 2:**
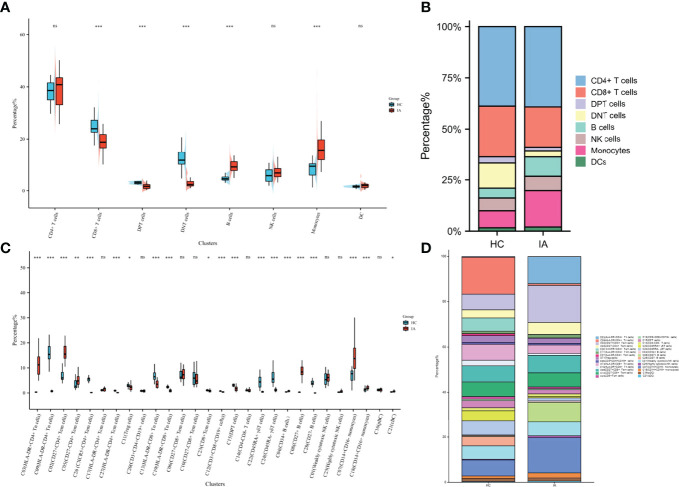
Changes in cell proportions in IA. **(A)** Percentage of 8 cell lineages in CD45^+^ cells from HC and IA patients. *P* values were calculated by the two-sided Wilcoxon test. Significance: ***p < 0.001; **p < 0.01; *p < 0.05; ns p ≥ 0.05. **(B)** Bar chart of the percentage of 8 cell lineages in CD45^+^ cells. **(C)** Percentage of 28 cell lineages in CD45+ cells from HC and IA patients. *P* values were calculated by the two-sided Wilcoxon test. Significance: ***p < 0.001; **p < 0.01; *p < 0.05; ns p ≥ 0.05. **(D)** Bar chart of the percentage of 28 cell lineages in CD45^+^ cells.

High-dimensional cytometry profiling was performed on PBMC samples isolated from 26 IA patients and 20 age- and sex-matched HC (**Figure 1**). The clinical and demographic features of IA patients and HCs are shown in **Table 1**. By CyTOF, we differentiated CD45^+^ PBMCs into 28 clusters based on the patterns of marker expression. These 28 cell clusters belonged to eight-cell lineages, including CD4^+^ T cells, CD8^+^ T cells, CD4^+^CD8^+^ double-positive T cells (DPT), CD4^-^CD8^-^ double-negative T cells (DNT), B cells, NK cells, dendritic cells (DCs), and monocytes (**Table 2**). Compared with those in the HC group, we found a higher proportion of B cells and monocytes in group IA, and a lower proportion of CD8+ T cells, DNT cells, and DPT cells. No significant difference was found in the frequencies of CD4^+^ T cells, NK cells, and DCs (**Figure 2**).

**Table 1:** Demographic and clinical characteristics in patients with IA.

Characteristics	Patients (n=26)	Controls (n=20)
Age	55.1 ± 15.5	55.4 ± 6.7
Female/male ratio	14/12	11/9
Vascular risk factors		
Hypertension	18 (69.2)	–
Hypercholesterolemia	1 (3.8)	–
Diabetes mellitus	2 (7.7)	–
Current cigarette smoking	8 (30.8)	5(25.0)
Current cigarette alcohol	3 (11.5)	2 (10.0)
Aneurysm location		
Anterior circulation	25 (96.2)	–
Posterior circulation	1 (3.8)	–
Single aneurysm	19 (73.1)	–
Multiple aneurysm	7 (26.9)	–

**Table 2:** Comparison of PhenoGraph clusters with manually gated cell phenotypes.

Cell lineages	Cell type	PhenoGraph cluster	Clusters
CD4^+^ T cells	CD4^+^ Tn	C03	HLA-DR^+^CD4^+^ Tn cells
		C09	HLA-DR^-^CD4^+^ Tn cells
	CD4^+^ Tem	C02	CD27^+^CD4^+^ Tem cells
		C05	CD27^-^CD4^+^ Tem cells
		C26	CXCR5^+^CD4^+^ Tem cells
	CD4^+^ Tcm	C17	HLA-DR^+^CD4^+^ Tcm cells
		C27	HLA-DR^-^CD4^+^ Tcm cells
	CD4^+^CD19^+^	C20	CD3+CD4^+^CD19^+^ cells
	CD4^+^CD25^+^	C11	Treg cells
CD8^+^ T cells	CD8^+^ Tn	C13	HLA-DR^-^CD8^+^ Tn cells
		C19	HLA-DR^+^CD8^+^ Tn cells
	CD8^+^ Tem	C06	CD27^+^CD8^+^ Tem cells
		C10	CD27^-^CD8^+^ Tem cells
	CD8^+^ Tcm	C23	CD8^+^ Tcm cells
	CD8^+^CD19^+^	C12	CD3^+^CD8^+^CD19^+^ cells
DPT cells	CD4^+^CD8^+^	C15	DPT cells
DNT cells	CD4^-^CD8^-^	C14	CD4^-^CD8^-^ cells
	CD45RA^+^ γδT	C22	CD45RA^+^ γδT cells
	CD45RA^-^ γδT	C24	CD45RA^-^ γδT cells
B cells	CD34^+^	C04	CD34^+^ B cells
	CD27^+^	C08	CD27^+^ B cells
	CD27^-^	C28	CD27^-^ B cells
NK cells	CD56^high^CD16^+^	C01	Weakly cytotoxic NK cells
	CD56^low^ CD16^-^	C25	Highly cytotoxic NK cells
Monocytes	CD14^+^CD16^-^	C07	CD14^+^CD16^-^ monocytes
	CD14^+^CD16^+^	C18	CD14^+^CD16^+^ monocytes
DC	CD11c^low^ CD123^+^	C16	Plasmacytoid DCs
	CD11c^+^ HLA-DR^+^	C21	Classical DCs

### Dysregulation of CD4^+^ T-Cell Clusters in IA Patients

**Figure 3:**
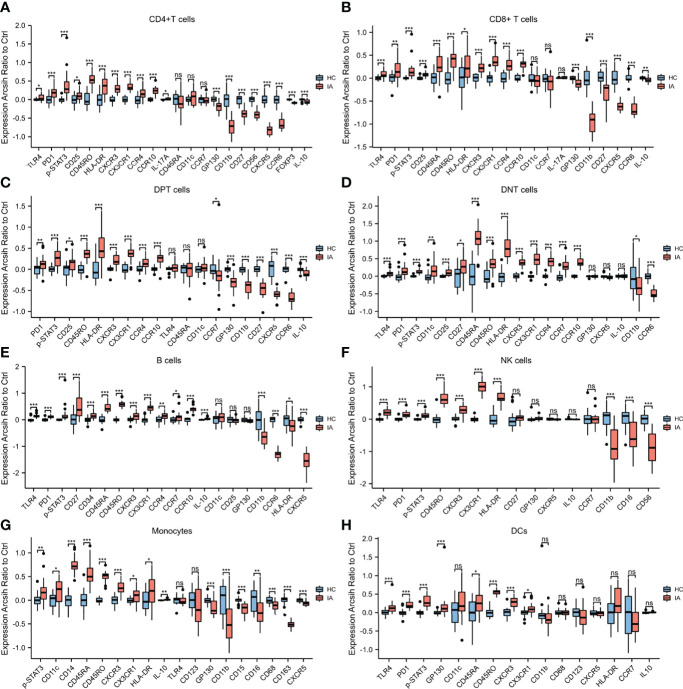
Marker expressions in 8 cell lineages. **(A)** Marker expressions in CD4+ T cells from HC and IA patients. The expression levels of TLR4, PD1, p-STAT3, CD25, CD45RO, HLA-DR, CXCR3, CX3CR1, CCR4, CCR10, and IL-17A in CD4+ T cells were higher in the IA group than in the HC group. The levels of GP130, CD11b, CD27, CD56, CXCR5, CCR6, FOXP3, and IL-10 were lower in the patient groups than in the HC group. **(B)** Marker expressions in CD8+ T cells from HC and IA patients. The expression levels of TLR4, PD1, p-STAT3, CD25, CD45RA, CD45RO, HLA-DR, CXCR3, CX3CR1, CCR4, and CCR10 in CD8+ T cells were higher in the IA group than in the HC group. However, the expression levels of GP130, CD11b, CD27, CXCR5, CCR6, and IL-10 were lower in the IA group than in the HC group. **(C)** Marker expressions in DPT cells from HC and IA patients. The expression of PD1, p-STAT3, CD25, CD45RO, HLA-DR, CXCR3, CX3CR1, CCR4, and CCR10 in DPT cells was higher in the IA group than in the HC group. The levels of GP130, CD11b, CD27, CXCR5, CCR6, and IL-10 were lower in the patient groups than in the HC group **(D)** Marker expressions in DNT cells from HC and IA patients. The expression levels of TLR4, PD1, p-STAT3, CD11c, CD25, CD27, CD45RA, CD45RO, HLA-DR, CXCR3, CX3CR1, CCR4, CCR7, and CCR10 in DNT cells were higher in the IA group than in the HC group. In addition, the levels of CD11b and CCR6 were lower in the patient groups than in the HC group.**(E)** Marker expressions in B cells from HC and IA patients. The expression levels of TLR4, PD1, p-STAT3, CD27, CD34, CD45RA, CD45RO, CXCR3, CX3CR1, CCR4, CCR7, CCR10, and IL-10 in B cells were higher in the IA group than in the HC group. The levels of CD11b, CCR6, HLA-DR, and CXCR5 were lower in the patient groups than in the HC group. **(F)** Marker expressions in NK cells from HC and IA patients. The expression levels of TLR4, PD1, p-STAT3, CD45RO, CXCR3, CX3CR1, and HLA-DR in NK cells were higher in the IA group than in the HC group. The levels of CD11b, CD16, and CD56 were lower in the IA group than in the HC group. **(G)** Marker expressions in monocytes from HC and IA patients. The expression levels of p-STAT3, CD11c, CD14, CD45RA, CD45RO, CXCR3, CX3CR1, and HLA-DR in monocytes were higher in the IA group than in the HC group. However, the levels of GP130, CD11b, CD15, CD16, CD68, CD163, and CXCR5 were lower in the IA group than in the HC group. **(H)** Marker expressions in DCs from HC and IA patients. The expression levels of TLR4, PD1, p-STAT3, GP130, CD11c, CD45RA, CD45RO, CXCR3, and CX3CR1 in DC cells were higher in the IA group than in the HC group. *P* values were calculated by the two-sided Wilcoxon test. Significance: ***p < 0.001; **p < 0.01; *p < 0.05; ns p ≥ 0.05.

Although no significant difference in the proportion of CD4^+^T cells was observed between the IA and HC groups, the expression levels of TLR4, PD1, p-STAT3, CD25, CD45RO, HLA-DR, CXCR3, CX3CR1, CCR4, CCR10, and IL-17A in CD4^+^T cells were higher in the IA group than in the HC group. The levels of GP130, CD11b, CD27, CD56, CXCR5, CCR6, FOXP3, and IL-10 were lower in the patient groups than in the HC group (**Figure 3A** **)**.

**Figure 4:**
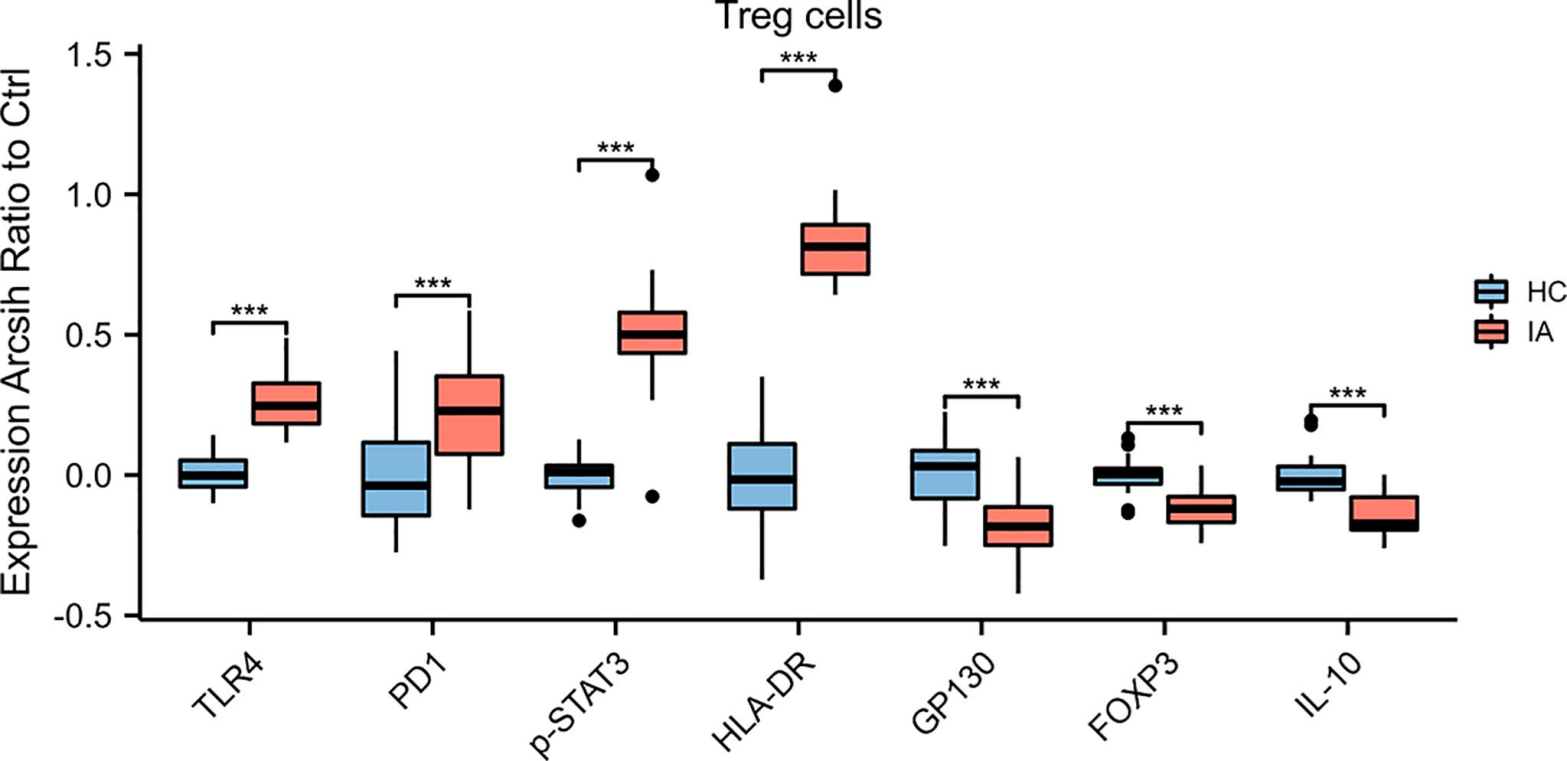
Marker expressions in Treg cells. The expression levels of TLR4, PD1, p-STAT3, and HLA-DR in Treg cells were higher, while those of GP130, Foxp3, and IL-10 were lower, in IA patients compared with HCs. *P* values were calculated by the two-sided Wilcoxon test. Significance: ***p < 0.001.

Among the 9 clusters (C02, C03, C05, C09, C11, C17, C20, C26, C27) of CD4^+^ T cells, eight clusters significantly differed between the IA and HC groups. Among the nine clusters, the proportions of C02 (CD27^+^CD4^+^ Tem), C03(HLA-DR^+^CD4^+^ Tn), C05(CD27^-^CD4^+^ Tem), and C17 (HLA-DR^+^CD4^+^ Tcm) were increased in the IA group compared with those in HCs, whereas C09(HLA-DR^-^CD4^+^ Tn), C11(Treg cells), C26(CXCR5^+^CD4^+^ Tem), and C27(HLA-DR^-^CD4^+^ Tcm) were decreased. No significant differences in the proportions of C20 (CD3^+^CD4^+^CD19^+^ cells) were observed between the IA and HC groups. Moreover, the expression levels of TLR4, PD1, p-STAT3, and HLA-DR in Treg cells were higher in IA patients, while those of GP130, Foxp3, and IL-10 were lower in IA patients compared with HCs (**Figure 4**).

### Lower Proportions of CD8^+^T Cell Clusters in IA Patients

Compared with the HC group, the proportions of CD8^+^T cells were significantly lower in the IA group. Furthermore, the expression levels of TLR4, PD1, p-STAT3, CD25, CD45RA, CD45RO, HLA-DR, CXCR3, CX3CR1, CCR4, and CCR10 in CD8^+^T cells were higher in the IA group than in the HC group. However, the expression levels of GP130, CD11b, CD27, CXCR5, CCR6, and IL-10 were lower in the IA group than in the HC group (**Figure 3B**).

Among six clusters (C06, C10, C12, C13, C19, C23) of CD8^+^ T cells, four clusters were significantly different between the IA and HC groups. Specifically, the proportions of C12(CD3^+^CD8^+^CD19^+^cells), C13(HLA-DR^-^CD8^+^Tn), CD19(HLA-DR^+^CD8^+^Tn), and C23(CD8^+^Tcm) were lower in the IA group than in the HC group. There was no difference in the proportions of C06 (CD27^+^CD8^+^Tem) or C10(CD27^-^CD8^+^Tem) between the IA and HC groups.

### The Proportions of DPT and DNT-Cell Clusters Were Lower in the IA Group

Compared with the HC group, the proportions of DPT (C15) were significantly lower in the IA group. Furthermore, the expression of PD1, p-STAT3, CD25, CD45RO, HLA-DR, CXCR3, CX3CR1, CCR4, and CCR10 in DPT cells was higher in the IA group than in the HC group. The levels of GP130, CD11b, CD27, CXCR5, CCR6, and IL-10 were lower in the patient groups than in the HC group (**Figure 3C**).

Compared with the HC group, the proportions of DNT cells were significantly decreased in the IA group. Furthermore, the expression levels of TLR4, PD1, p-STAT3, CD11c, CD25, CD27, CD45RA, CD45RO, HLA-DR, CXCR3, CX3CR1, CCR4, CCR7, and CCR10 in DNT cells were higher in the IA group than in the HC group. In addition, the levels of CD11b, and CCR6 were lower in the patient groups than in the HC group (**Figure 3D**). Among the three clusters (C14, C22, C24) of DNT cells, two clusters were significantly different between the IA and HC groups. Compared with the HC group, the proportions of C22 (CD45RA^+^ γδT), and C24(CD45RA^-^ γδT) were decreased in the IA group.

### B-Cell and NK-Cell Clusters in the IA Group

Patients in the IA group had a higher percentage of B cells than HCs. Furthermore, the expression levels of TLR4, PD1, p-STAT3, CD27, CD34, CD45RA, CD45RO, CXCR3, CX3CR1, CCR4, CCR7, CCR10, and IL-10 in B cells were higher in the IA group than in the HC group. The levels of CD11b, CCR6, HLA-DR, and CXCR5 were lower in the patient groups than in the HC group (**Figure 3E**). Among the three clusters (C04, C08, C28) of B cells, we found that the proportions of C04(CD34^+^ B cells), and C08(CD27^+^B cells) were higher, C28(CD27^-^ B cells) were lower in the IA group than in the HC group.

There were no differences in the proportions of NK cells between the IA and HC groups. Furthermore, the expression levels of TLR4, PD1, p-STAT3, CD45RO, CXCR3, CX3CR1, and HLA-DR in NK cells were higher in the IA group than in the HC group. The levels of CD11b, CD16, and CD56 were lower in the IA group than in the HC group (**Figure 3F**). Neither C01(highly cytotoxic NK cells) nor C25 (weakly cytotoxic NK cells) was significantly different between groups.

### Monocytes and DC Clusters in the IA Group

Patients in the IA group had an increased percentage of monocytes compared with the HC group. Furthermore, the expression levels of p-STAT3, CD11c, CD14, CD45RA, CD45RO, CXCR3, CX3CR1, and HLA-DR in monocytes were higher in the IA group than in the HC group. However, the levels of GP130, CD11b, CD15, CD16, CD68, CD163, and CXCR5 were lower in the IA group than in the HC group (**Figure 3G**). In addition, the proportions of C07(CD14^+^CD16^-^monocytes) and C18(CD14^+^CD16^+^ monocytes) were significantly higher in the IA group than in the HC group.

Compared with the HC group, the proportions of DC were not significantly increased in the IA group. Furthermore, the expression levels of TLR4, PD1, p-STAT3, GP130, CD11c, CD45RA, CD45RO, CXCR3, and CX3CR1 in DC cells were higher in the IA group than in the HC group (**Figure 3H**). The proportions of C21(classical DC) were significantly higher in the IA group than in the HC group, and the percentage of C16 (plasmacytoid DC) in the IA group was not different from that in the HC group.

## Discussion

IA is increasingly recognized as a disease driven by chronic inflammation (5, 7, 14, 15). Circulating immune cells may contribute to IA formation and progression by infiltrating the vessel walls, releasing proinflammatory molecules, mediating the destruction of the endothelium and the extracellular matrix and promoting the pathogenic proliferation of smooth muscle cells (10, 16, 17). In this study, we demonstrated that IA patients exhibited dysfunction of immunity, with dysregulation of CD4^+^ T-cell clusters, increased B cells and monocytes, and decreased CD8^+^ T cells, DNT cells, and DPT cells. Moreover, compared with findings in HCs, IA was associated with enhanced lymphocyte and monocyte immune activation, with a higher expression of HLA-DR, CXCR3, and CX3CR1.

Dysregulation of CD4^+^ T cells is involved in the pathogenesis of various chronic inflammatory disorders. A previous study demonstrated that IA patients exhibited an imbalance of CD4^+^ T cell subsets (11, 18–20). Zhang et al. found that the frequencies Th1 and Th17 cells in IA patients were higher, and the frequencies of Th2 and Treg were lower than those in HCs (11). Song et al. also demonstrated an imbalance in circulating Th17/Treg in IA patients, and the proportions of Th17 were associated with the severity of IA-induced SAH (9). In this study, the imbalance of CD4^+^ T cell subsets in IA patients was further confirmed, with higher frequencies of C02 (CD27^+^CD4^+^ Tem), C03(HLA-DR^+^CD4^+^ Tn), C05(CD27^-^CD4^+^ Tem), and C17 (HLA-DR^+^CD4^+^ Tcm), and lower frequencies of C09(HLA-DR^-^CD4^+^ Tn), C11(Treg cells), C26(CXCR5^+^CD4^+^ Tem), and C27(HLA-DR^-^CD4^+^ Tcm). In addition, recent studies have shown that Treg cells were significantly decreased in IA patients compared with HCs (9, 20–22) Additionally, the expression leves of Foxp3, and IL-10 were significantly lower in Treg cells from IA patients than from HCs (20–22), which is also consistent with our findings.

Cytotoxic immune cells play a crucial role in inflammation. There are two types of cytotoxic cells: one is involved in innate or natural immunity (γδT cells, NK cells, NKT cells), and the other is involved in acquired immunity (CD8^+^T cells) (23). In our study, we found that the proportions of cytotoxic cells, including CD8^+^T cells, and γδT cells, were decreased in patients with IA. Moreover, a recent study also revealed a decreased proportions of circulating CD8^+^T cells in IA patients (24, 25). Although the number of cytotoxic immune cells is decreased in IA patients, the migration capacity of cells is enhanced, with a higher expression of CXCR3 and CX3CR1 in CD8^+^T cells, γδT cells, and NK cells.

B cells play a key role in the pathogenesis of chronic inflammation (26). Along with the ability to produce antibodies, B cells can contribute to the development of disease by presenting autoantigens to autoreactive T cells and by secreting proinflammatory cytokines and chemokines that amplify the inflammatory response (27). In patients with abdominal aortic aneurysms (AAAs), B cells are abundant, and depletion of B cells can prevent experimental AAA formation in mice (28). Although the importance of circulating B cells in IA is not well-documented, several studies have shown that humoral immunity plays a role in IA formation (29). In our study, the proportions of C04(CD19^+^CD34^+^cells), and C08(CD27^+^B cells) were higher, while that of C28(CD27^-^ B cells) was lower in the IA group than in the HC group, which suggested that circulating activated B cells may play a critical role in IA.

In addition to lymphocytes, circulating monocytes may play a role in the progression of damage in IA by leaving the bloodstream, migrating through the endothelium, and differentiating into tissue macrophages, and these tissue macrophages can prime and shape further immune responses (10, 30). A previous study revealed that the proportions of CD14^+^CD16^+^ and CD14^dim^CD16^+^ monocytes were higher in IA patient than in HCs, whereas, that of CD14^+^CD16^−^ monocytes were lower decreased (10). In this study, we found that the frequencies of both CD14^+^CD16^+^ and CD14^+^CD16^-^ were higher in IA patients than in HCs. In addition, the higher expression of CD11c, CXCR3, and CX3CR1 in IA patient monocytes revealed that monocyte adhesion and chemokine capacity increased.

Although DCs are the major APCs and play a pivotal role in the initiation and activation of the immune response (31), few studies have focused on DCs in patients with IA. In experimental AAA, depletion of CD11c^+^ DCs limits AAA formation and progression (32). The depletion of CD11c^+^ DCs may be related to a decline in the proportion of circulating activated T and B cells, and reduced matrix metalloproteinase activity in the vascular wall (32). Another speculation is that DCs may be involved in aneurysm formation through damage-associated molecular patterns (29). In this study, we found that the proportions of C21(classical DCs) and the expression of CD11c were significantly higher in the IA group than in the HC group, which is consistent with a previous study on AAA. Moreover, the significantly increased expressionof CXCR3, and CX3CR1 in DCs suggested that the capacity of inflammatory cells to migrate was increased.

In the current study, the expression of TLR4 in circulating immune cellswas widely enhanced. As a member of the TLR family, TLR4 plays a crucial role in innate and adaptive immunity processes, which are involved in the pathogenesis of many inflammatory disorders (33). In previous studies, TLR4 was significantly associated with susceptibility to IA and expressed in IA walls (34–36). Furthermore, the TLR4 pathway promotes the development of IA rupture by accelerating inflammation in aneurysmal walls (34). In addition, TLR4 was also found to activate NF-κB, increasing the release of proinflammatory cytokines and chemokines (37). NF-κB is a key mediator of IA formation by inducing some inflammatory genes related to macrophage recruitment and activation (38, 39). Inhibiting the TLR4 pathway in inflammatory cells may be a promising approach to preventing IA formation and rupture (34).

In addition to TLR4, we also found that the expression of p-STAT3 in circulating immune cells was widely enhanced. STAT3 plays a vital role during inflammatory responses (40). Previous studies demonstrated that STAT3 is a key regulator in IA formation by modulating inflammatory cytokine expression (41). The expression of STAT3 was increased in ruptured IA tissues and closely correlated with IA diameter and IA type (41). Furthermore, the inhibition of STAT3 in inflammatory cells prevented IA formation and rupture (42). STAT3 may be a potential therapeutic target in IA formation and rupture. HLA-DR expression on T cells is regarded as a marker of T-cell activation (43), and T-cell activation leads to increased expression of PD1 proteins on the surface of T cell. And PD1 is a key immune checkpoint receptor that expressed on activated T-cells to trigger immune tolerance (44). In this study, we found that both PD1 and HLA-DR were highly upregulated in T cells, including CD4^+^T, CD8^+^T, DPT, and DNT cells, which suggested widespread activation of T cells in IA. However, no studies concerning PD1 in IA have been reported. However, inhibiting the expression of PD1 can decrease AAA formation in mouse and rat models (45), suggesting that PD1 may be a molecular target for IA therapy.

There are several limitations of this study. First, the sample size was small and patients were from a single-center, the study may have selection bias. Second, our study did not further verify the function of immune cells through cell or animal experiments. Third, only patients with unruptured IA were enrolled in our study, and this study could not demonstrate the circulating immune cell landscape of ruptured IA. Fourth, comorbidities, such as hypertension, hypercholesterolemia, and diabetes may affect circulating immune cells. All control subjects enrolled in the current study were healthy, and only age and sex matched, and they had few comorbidities. In future studies, we will select the interesting clusters to verify the mechanisms of changes in circulating immune cells in IA, and enroll the patients with ruptured IA to determine the changes in the circulating immune cell landscape in ruptured IA.

## Conclusions

In summary, our data provide an overview of the circulating immune cell landscape in IA and reveal that the dysfunction of circulating immunity may play a potential role in the development of IA.

## Data Availability Statement

The raw data supporting the conclusions of this article will be made available by the authors, without undue reservation.

## Ethics Statement

The studies involving human participants were reviewed and approved by Ethics Committee of Beijing Tiantan Hospital, Capital Medical University. The patients/participants provided their written informed consent to participate in this study.

## Author Contributions

PG, WW, DZ and JZ: conception and design. CL, LC, YP, HL, and JW: acquisition of data. PG, and WW: analysis and interpretation of data. PG: drafting the article. QZ, XY, RW, YZ and DZ: technical supports. All authors critically revising the article and approved the final version of the manuscript. JZ: study supervision. All authors contributed to the article and approved the submitted version.

## Funding

This study was supported by National Natural Science Foundation of China (81701137 and 81870904).

## Conflict of Interest

The authors declare that the research was conducted in the absence of any commercial or financial relationships that could be construed as a potential conflict of interest.

## Publisher’s Note

All claims expressed in this article are solely those of the authors and do not necessarily represent those of their affiliated organizations, or those of the publisher, the editors and the reviewers. Any product that may be evaluated in this article, or claim that may be made by its manufacturer, is not guaranteed or endorsed by the publisher.
