# Supplementary material for: High-Dimensional Immune Profiling by Mass Cytometry Revealed the Circulating Immune Cell Landscape in Patients With Intracranial Aneurysm
Source: Front Immunol. 2022 Jun 27;13:922000. doi: 10.3389/fimmu.2022.922000 (PMC9271834; doi:10.3389/fimmu.2022.922000)
Supplement: Supplementary file 1 [file Table_1.docx]

Supplementary Material

**Supplementary Table 1. Mass cytometry antibodies panel design.**

| Antigen | Symbol and Mass | Antibody clone | Source |
| --- | --- | --- | --- |
| CD45 | 89Y | HI30 | Fluidigm |
| CCR6 | 141Pr | G034E3 | Fluidigm |
| TLR4 | 142Ce | 76B357.1 | Abcam |
| CD45RA | 143Nd | HI100 | Fluidigm |
| CCR10 | 144Nd | 314305 | R&D |
| CD16 | 145Nd | 3G8 | Fluidigm |
| CD8 | 146Nd | RPA-T8 | Fluidigm |
| CD11c | 147Sm | Bu15 | Fluidigm |
| CD15 | 148Sm | ICRF29-2 | R&D |
| CD25 | 149Sm | BC96 | Fluidigm |
| CD27 | 150Sm | L128 | Fluidigm |
| GP130 | 151Eu | 28126 | R&D |
| TCRgd | 152Gd | 11F2 | Fluidigm |
| CXCR5 | 153Eu | RF8B2 | Fluidigm |
| CCR7 | 154Gd | G043H7 | R&D |
| PD1 | 155Gd | EH12.2H7 | Fluidigm |
| CXCR3 | 156Gd | G025H7 | Fluidigm |
| pSTAT3 | 158Gd | 4/P-STAT3 | Fluidigm |
| FOXP3 | 159Tb | PCH101 | Fluidigm |
| CD14 | 160Dy | 134620 | R&D |
| IL-17A | 161Dy | BL168 | Fluidigm |
| CD34 | 162Dy | EP373Y | Abcam |
| CD45 | 163Dy | HI30 | Abcam |
| CCR4 | 164Dy | EPR23502-85 | Abcam |
| CD45RO | 165Ho | UCHL1 | Fluidigm |
| IL-10 | 166Er | JES3-9D7 | Abcam |
| CD163 | 167Er | 215927 | R&D |
| CD56 | 168Er | NCAM16.2 | R&D |
| CD45 | 169Tm | HI30 | Abcam |
| CD3 | 170Yb | UCHT1 | Fluidigm |
| CD123 | 171Yb | 32703 | R&D |
| CX3CR1 | 172Yb | 528728 | R&D |
| CD68 | 173Yb | Y1/82A | R&D |
| HLA_DR | 174Yb | L243 | R&D |
| CD19 | 175Lu | HIB19 | R&D |
| CD4 | 176Lu | RPA-T4 | R&D |
| CD11b | 209Bi | ICRF44 | Fluidigm |
